# Structures of myxobacterial phytochrome revealed by cryo-EM using the Spotiton technique and with x-ray crystallography

**DOI:** 10.1063/4.0000301

**Published:** 2025-05-01

**Authors:** Prabin Karki, David Menendez, William Budell, Shishir Dangi, Carolina Hernandez, Joshua Mendez, Srinivasan Muniyappan, Shibom Basu, Peter Schwander, Tek N. Malla, Emina A. Stojković, Marius Schmidt

**Affiliations:** 1 Department of Physics, University of Wisconsin-Milwaukee, Milwaukee, Wisconsin 53211, USA; 2 Department of Biology, Northeastern Illinois University, Chicago, Illinois 60625, USA; 3 New York Structural Biology Center (NYSBC), New York, New York 10027, USA; 4 European Molecular Biology Laboratory (EMBL), Grenoble, France

## Abstract

Phytochromes are red-light photoreceptors first identified in plants, with homologs found in bacteria and fungi, that regulate a variety of critical physiological processes. They undergo a reversible photocycle between two distinct states: a red-light-absorbing Pr form and a far-red light-absorbing Pfr form. This Pr/Pfr photoconversion controls the activity of a C-terminal enzymatic domain, typically a histidine kinase (HK). However, the molecular mechanisms underlying light-induced regulation of HK activity in bacteria remain poorly understood, as only a few structures of unmodified bacterial phytochromes with HK activity are known. Recently, cryo-EM structures of a wild-type bacterial phytochrome with HK activity are solved that reveal homodimers in both the Pr and Pfr states, as well as a heterodimer with individual monomers in distinct Pr and Pfr states. Cryo-EM structures of a truncated version of the same phytochrome—lacking the HK domain—also show a homodimer in the Pfr state and a Pr/Pfr heterodimer. Here, we describe in detail how structural information is obtained from cryo-EM data on a full-length intact bacteriophytochrome, and how the cryo-EM structure can contribute to the understanding of the function of the phytochrome. In addition, we compare the cryo-EM structure to an unusual x-ray structure that is obtained from a fragmented full-length phytochrome crystallized in the Pr-state.

## INTRODUCTION

Sensing and responding to changing environmental conditions is crucial for the survival of living organisms. In prokaryotes, environmental signals are typically detected through a two-component signaling system, consisting of a histidine kinase (HK) and a response regulator (RR) (Stock *et al.*, 2000). These highly modular two-component systems have been adapted into diverse cellular signaling circuits. In photosynthetic and non-photosynthetic bacteria, phytochrome photoreceptors are integrated into such signaling mechanisms, responding to variations in light spectrum, intensity, and direction (Furuya, 1993). Phytochromes are also found in fungi and were originally discovered in plants (Davis *et al.*, 1999; Rockwell *et al.*, 2006). Plant phytochromes, such as PhyA and PhyB, feature a C-terminal histidine kinase-like domain (HKLD), but their domain composition differs from that of bacterial phytochromes. Recent cryo-EM studies have revealed their unusual homodimeric structures (Li *et al.*, 2022; Zhang *et al.*, 2023). Upon light activation, plant Phys migrate to the nucleus where they induce responses such as germination, greening, and shade avoiding (Batschauer, 1998) through a mechanism distinct from the bacterial two-component system (Cheng *et al.*, 2021).

In photosynthetic bacteria, phytochromes regulate responses such as the synthesis of light-harvesting complexes (Hughes *et al.*, 1997; Evans *et al.*, 2005; Rockwell *et al.*, 2006; Rockwell *et al.*, 2009; and Burgie *et al.*, 2020). In contrast, their roles in non-photosynthetic bacteria are diverse (Hughes and Winkler, 2024), ranging from facilitating gene transfer in *Agrobacterium fabrum* (formerly *tumefaciens*) (Lamparter *et al.*, 2021) to controlling multicellular fruiting body formation in myxobacteria (Wagner *et al.*, 2005; Auldridge and Forest, 2011; and Woitowich *et al.*, 2018). Recent advances using single-particle cryo-EM have uncovered new structural details of the phytochrome-mediated two-component system in the non-photosynthetic myxobacterium *Stigmatella aurantiaca* (Sanchez *et al.*, 2019; Malla *et al.*, 2024). Myxobacteria are unique among prokaryotes for their multicellular stage, forming complex fruiting bodies. In *S. aurantiaca*, this process is regulated by red and far-red light, highlighting the role of bacteriophytochrome (BphP) signaling (Qualls *et al.*, 1978; White *et al.*, 1980; and Woitowich *et al.*, 2018). Additionally, myxobacteria are widely studied for their production of secondary metabolites with potential anticancer and antimicrobial applications, recently shown to be influenced by light (Schaberle *et al.*, 2014; Lapuhs *et al.*, 2022).

**FIG. 1.**
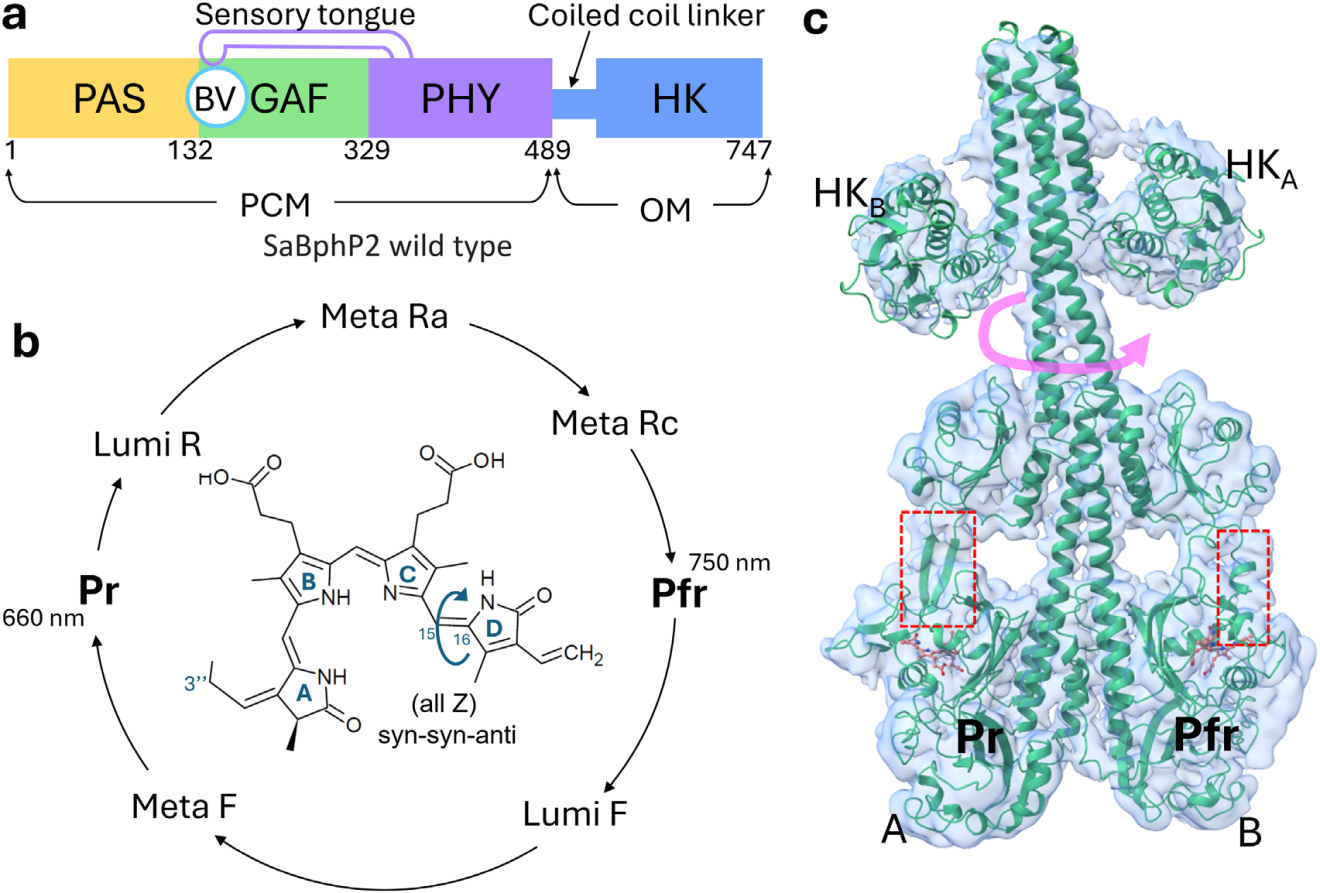
The *S. aurantiaca* BphP2. (a) Scheme of the domain architecture of the wild-type SaBphP2. Sequence numbers are marked. PAS, GAF, and PHY domains form the PCM which is covalently linked to the output effector module (OM). In SaBphP2, the OM is a histidine kinase (HK). The BV chromophore is covalently bound to the PAS domain. The BV binding pocket is shared with the GAF domain. The PHY domain has a long extension called the sensory tongue that is required for complete Pr/Pfr photoconversion. (b) The reversible photocycle with intermediates. When the Pr state is illuminated with red light (640–700 nm), it converts to the Pfr state. When illuminated with far-red light (750 nm), the Pfr state can be driven to Pr. The structure of BV is shown with the four pyrrole rings in the (all Z) syn–syn–anti configuration. The C_15_=C_16_ double bond between rings C and D that undergoes Z to E isomerization is marked by an arrow. (c) Structure of the full-length SaBphP2 Pr/Pfr heterodimer as determined by cryo-EM (Malla *et al.*, 2024). The linker crosses subunit boundaries. The conformations of the sensory tongues are marked with the red dashed boxes. The presence of the Pfr state is indicated by the separation of the two stands with one strand in the tell-tale α-helix conformation. When the second subunit, here subunit A, transitions to Pfr, the HK lobes rotate (red arrow).

BphPs exist as dimers where each strand of amino acids has the same chemical composition (sequence). BphPs consist of a photosensory core module (PCM) and an output effector module (OM) [Fig. 1(a)]. The PCM consists of three domains: PAS (Per-Arndt-Sim), GAF (cGMP phosphodiesterase/adenylyl cyclase/FhIA), and PHY (phytochrome-specific GAF-related). The central chromophore, biliverdin (BV) [Fig. 1(b)], is a heme-derived open-chain tetrapyrrole (consisting of pyrrole rings A–D) which is covalently bound via its 3″ position to a conserved N-terminal cysteine (Cys13). The C-terminal OM differs among BphPs. The HK domain is the most common (Rockwell *et al.*, 2006; Kojadinovic *et al.*, 2008; and Otero *et al.*, 2016). *In S. aurantiaca*, the two BphPs, SaBphP1 (Woitowich *et al.*, 2018) and SaBphP2 (Sanchez *et al.*, 2019), share the same domain composition, including a HK domain [Fig. 1(a)]. HK activity has been experimentally confirmed for SaBphP2 by Malla *et al.* (2024). Light-dependent HK activity has been demonstrated in other BphPs, such as *Rhodopseudomonas palustris* RpBphP2 and RpBphP3 (Giraud *et al.*, 2005). Additionally, certain BphPs exhibit alternative functions: for example, *Idiomarina sp.* IsPadC features a diguanylyl cyclase OM (Gourinchas *et al.*, 2017), while *Deinococcus radiodurans* DrBphP exhibits phosphatase activity (Wahlgren *et al.*, 2022).

BphPs undergo photoconversion between a red-light-absorbing Pr state and a far-red-light-absorbing Pfr state through a series of intermediates [Fig. 1(b)] (Auldridge and Forest, 2011). Upon illumination with red light (λ = 640 nm), the biliverdin (BV) chromophore isomerizes from an all-Z to a ZZE configuration. This isomerization involves the rotation of BV's ring D around the C15=C16 double bond located between pyrrole rings C and D [Fig. 1(b)], initiating the first half of the photocycle (Takala *et al.*, 2014; Burgie *et al.*, 2016; Carrillo *et al.*, 2021; and Lopez *et al.*, 2022). The sensory tongue of the PHY domain [Figs. 1(a) and 1(c)] shifts between a β-strand conformation in the Pr and an α-helical conformation in the Pfr state as demonstrated by several structural and spectroscopic studies (Takala *et al.*, 2014; Bjorling *et al.*, 2015; Burgie *et al.*, 2016; Lopez *et al.*, 2022; Wahlgren *et al.*, 2022; Burgie *et al.*, 2024; and Malla *et al.*, 2024).

In intact BphPs, the signal is transmitted over approximately 100 Å from the BV to the output module (OM). The mechanism by which the light-regulated HK activity is controlled finally is coming to light with the first near-atomic resolution structures of a wild-type BphP with HK activity revealed by single-particle cryo-EM (Malla *et al.*, 2024). A sequence of amino acid residues known as the PRXSF motif (Essen *et al.*, 2008; Stojkovic *et al.*, 2014), located in the sensory tongue within the PHY domain, plays a pivotal role in the Pr-to-Pfr transition and, consequently, in light sensing. In the dark-adapted Pr state, the Arg residue of the PRXSF motif forms a key salt bridge with the Asp residue of the PASDIP motif (Yang *et al.*, 2007) in the GAF domain, which houses the BV chromophore [Fig. 1(a)]. After BV isomerization occurs in one of the subunits, the corresponding Arg-Asp salt bridge is disrupted, caused by the rotation of BV ring-D during isomerization. The two β-strands of the sensory tongue separate. One of the strands transitions to a α-helix [Fig. 1(c)]. The β-strand to an α-helix transition causes relative domain reorientations in the PCM but not in the HK domain. These structural reorientations result in the formation of a kink in the linker region of the other subunit, which is still in the Pr-state. This results in a Pr/Pfr heterodimer (Malla *et al.*, 2024). The signaling state is reached by photo-activating also the second subunit. A kink is now formed in the linker region of the former subunit and, as a consequence, the HK lobes rotate by about 60° relative to their positions observed in both the Pr/Pfr heterodimer and the Pr homodimer [see arrow in Fig. 1(c)].

The Pr to Pfr transition has a decisive impact on the HK enzymatic activity with the Pfr state typically displaying a lower activity (Wahlgren *et al.*, 2022; Kumarapperuma *et al.*, 2023; and Malla *et al.*, 2024). BphPs are a part of a two-component signaling pathway involving a response regulator (RR) protein (Stock *et al.*, 2000; Gao *et al.*, 2019). The HK enzyme first self-phosphorylates the BphP and, when the RR is present, transfers the phosphate to the RR (Kumarapperuma *et al.*, 2023). Association of the RR to and dissociation from the BphP is likely impacted by the strong rotation of the HK domain after photoactivation. In myxobacteria, activated RR proteins seem to control swarming and fruiting body formation (Graniczkowska *et al.*, 2024).

Crystal structures of the SaBphP1 and SaBphP2 PCMs in the Pr state provided the first insight into light-sensing enzymes in myxobacteria (Woitowich *et al.*, 2018; Sanchez *et al.*, 2019). Both proteins crystallized as homodimers, similar to the PCM crystal structures of DrBphP in Pr and Pfr states (Takala *et al.*, 2014; Burgie *et al.*, 2016), the bathy phytochrome PaBphP with Pfr as the dark-adapted state(Yang *et al.*, 2008) and plant PhyB in the Pr state (Burgie *et al.*, 2014). Remarkably, a Pr/Pfr heterodimer that had eluded previous studies was identified in cryo-EM structures of the intact (full-length) SaBphP2 (Malla *et al.*, 2024). Notably, a modified full-length BphP from *Idiomarina* sp. was also resolved as a Pr/Pfr heterodimer using crystallographic methods; however, this required the introduction of mutations in the linker region between the PCM and the diguanylyl cyclase domain (Tran *et al.*, 2024). In the absence of light, the photocycles of several BphPs revert thermally to the Pr state at varying rates, from minutes to hours, making crystallization of the Pfr state challenging (Burgie *et al.*, 2016).

In the recent single-particle cryo-EM experiment on the full-length SaBphP2 (Malla *et al.*, 2024), the density for the HK domain has been weak except for the Pr/Pfr heterodimer although the density of two HK moieties was good enough that the orientations of the HK lobes relative to the other domains could be determined. In order to improve the representation of the HK lobes also in the Pr/Pr homodimer, we used the Spotiton technique (Dandey *et al.*, 2020; Budell *et al.*, 2021) to prepare grids for the cryo-EM experiments. Here, we report details of cryo-EM density map generation and structure solution. In addition, we demonstrate how crystals of a severely truncated fragment of SaBphP2 can be obtained from which an x-ray structure with a very unusual chromophore configuration is determined.

## MATERIALS AND METHODS

### SaBphP2 preparation

Full-length SaBphP2 with an N-terminal 6His-tag is expressed in *E. coli* as described (Malla *et al.*, 2024). The construct can be purified in one step by using a Talon Co^+2^ metal ion affinity chromatography column, stored in stabilization buffer (20 mmol/l NaCl, 20 mmol/l Tris–HCl, and pH 8.0) at a concentration of about 10 mg/ml and frozen immediately. The frozen solution is shipped to New York Structural Biology Center (NY-SBC).

### Cryo-EM grid preparation

The pure Pr state is produced by pre-illuminating the sample at a concentration of 0.4 mg/ml with 740 nm light. The solution is loaded under green safety light into the sample cup of the Spotiton setup, which is described in detail in (Budell *et al.*, 2021). A (self-wicking) nanowire grid was mounted and lowered toward the ethene bath with an acceleration of 10 m/s^2^. 50–70 pl droplets (3.5 nl) of the photochrome solution was sprayed for 4 ms onto the grid during the initial acceleration. The grid is frozen ∼150 ms after.

### Cryo-EM imaging

The grids are imaged by a Thermo Fisher Scientific Titan Krios microscope equipped with a Gatan BioQuantum K3 energy filter direct electron detector camera. Movies with SaBphP2 in the pure Pr state are collected in the counting mode with a 2500 ms exposure time, 50 frames, 50 ms per frame, and a total dose of 45.47 e^−^/Å^2^. A total of 20 362 movies are collected at a pixel size of 0.829 Å/px. The individual frames of the movies are aligned (motion corrected) by using the program MotionCor2 v1.1.0 (Li *et al.*, 2013) and combined into micrographs by NYSBC. The micrographs are then transferred to the University of Wisconsin Milwaukee (UWM) via Globus Connect (www.globus.org). The transfer of data requires several days.

### Data processing

**FIG. 2.**
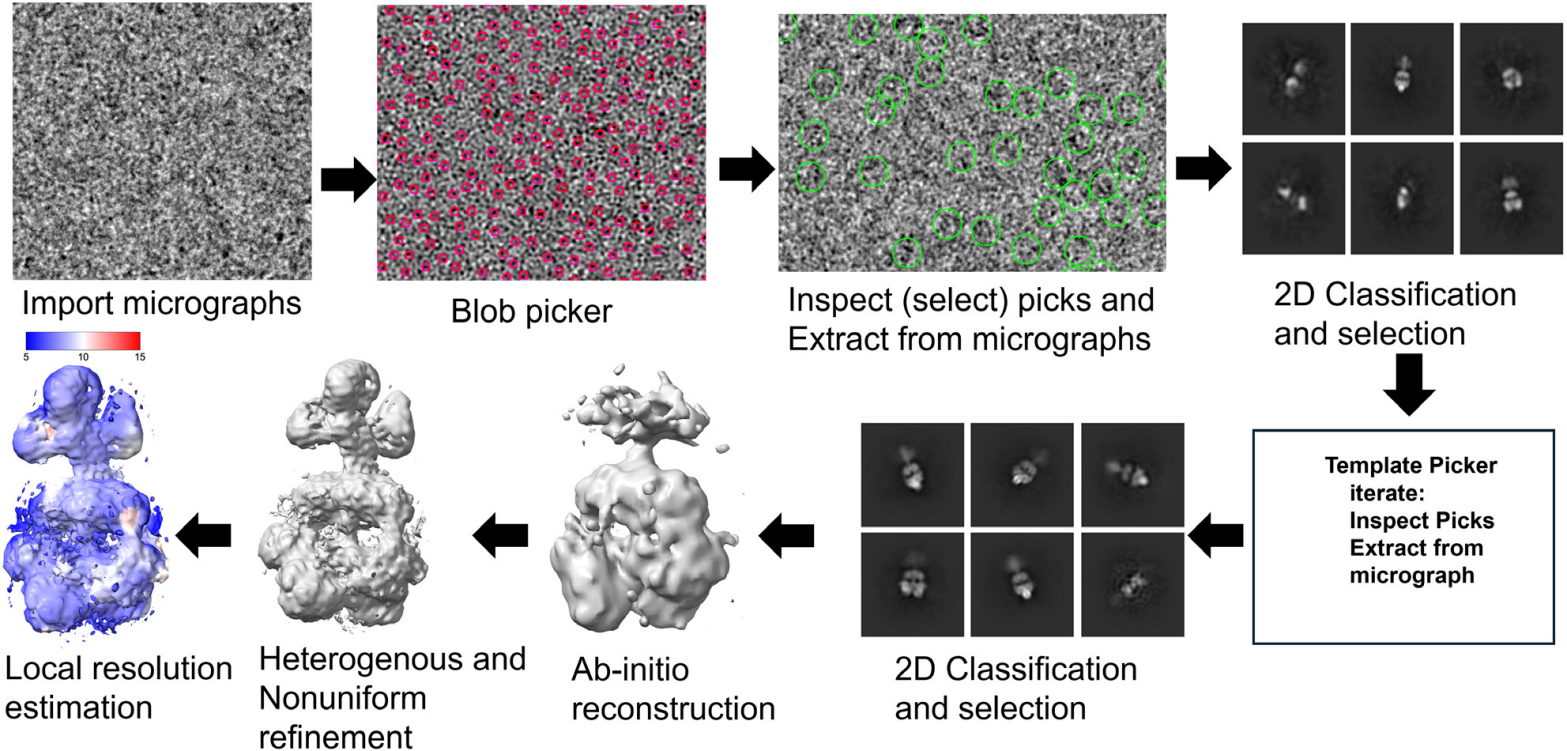
Bootstrapping cryoSPARC data processing sequence, see text and Table I.

**FIG. 3.**
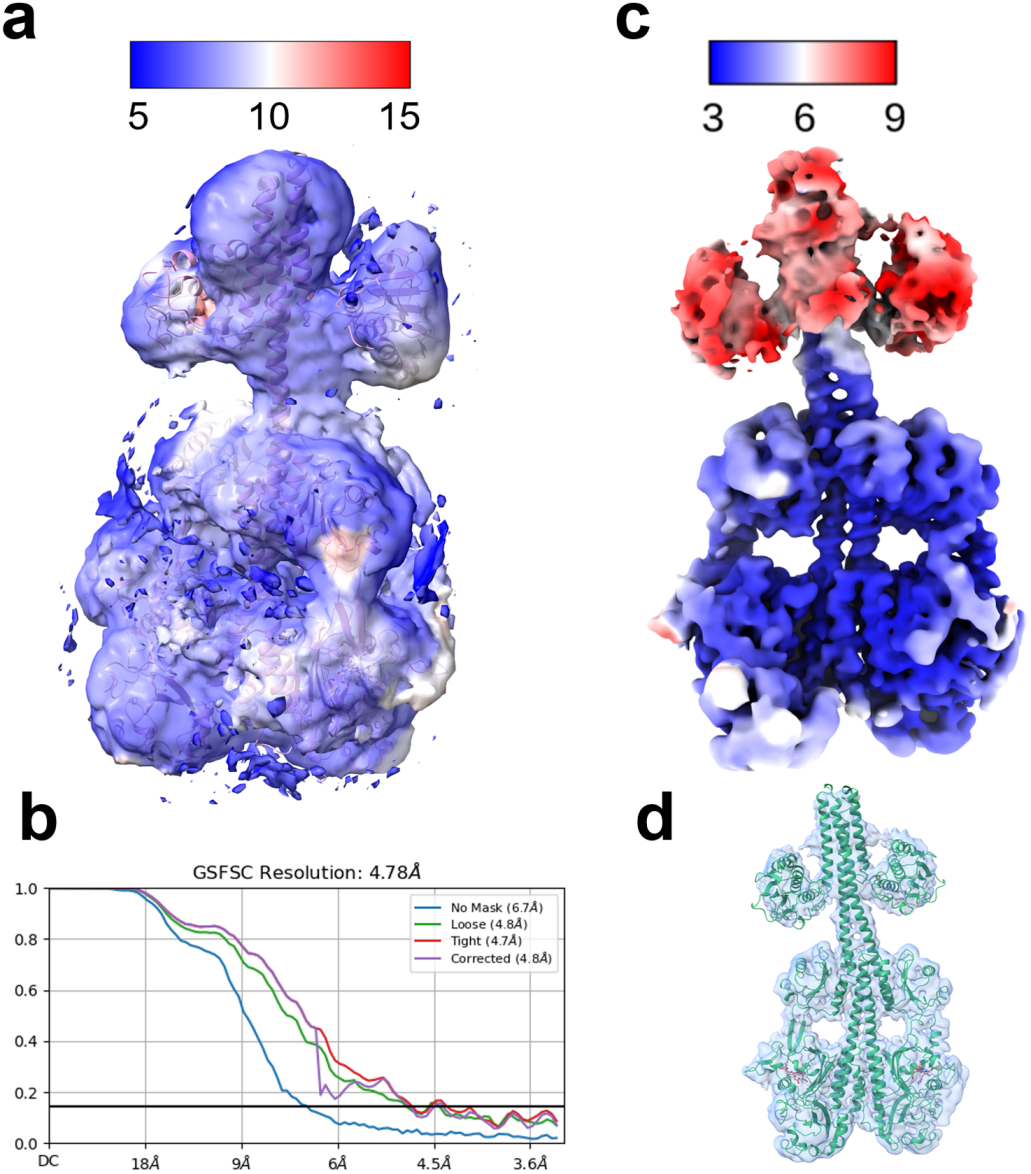
The structure of the full-length SaBphP2 determined by cryo-EM. (a) The structure of the Pr/Pr homodimer as reported here. The cryo-EM map is represented as a local resolution map. The resolution (in Å) is indicated by the scale bar. The atomic structure of the Pr/Pr homodimer is shown by the blue ribbon. (b) Fourier shell analysis computed by cryoSPARC of the Pr/Pr data from (a). (c) The local resolution map of the Pr/Pfr heterodimer as determined by Malla *et al.* (2024). (d) Atomic structure of the Pr/Pfr heterodimer as fit into the cryo-EM density map (blue).

The micrographs need to be curated to be able to pick appropriate particle images that then can be combined to a 3D cryo-EM map. This requires several steps that are all executed with cryoSPARC (Punjani *et al.*, 2017) installed on the High Performance Compute Cluster at UWM. The steps are explained in depth in the cryoSPARC tutorial. Figure 2 graphically shows the progress of data processing, which is described in the following. The motion-corrected micrographs received from NYSBC are processed using the microscope parameters for data processing as provided by NYSBC, which are listed in Table I. The contrast transfer function (CTF) is estimated by “patch CFT estimation” in cryoSPARC. The patch CTF estimation process divides a micrograph into multiple patches and creates a defocus landscape of the micrograph, which is used to estimate the CTF. The CTF correction is applied later at the step of 2D classification. Since the images can be crowded and can contain ice contamination and other artifacts such as fragmented particles, the images have to be curated, initially by hand, which is mainly determining outliers due to ice thickness. This is implemented in cryoSPARC as “manually curate exposure” (see Table I for thresholds). As a result, 4911 micrographs are rejected. From the remaining, curated micrographs, particles are picked by using the “blob-picker” in CryoSPARC using particle diameters from 70 to 220 Å. The remaining parameters are kept as default. 21 860 817 blobs or particles are picked. The number of picked particles now has to be reduced by selecting “promising” or “good” particles based on the rational assumption. For this, cryoSPARC provides a routine called “inspect particle peaks.” Good particles correlate well with other good particles. Conversely, sharp edges, such as ice crystal and other aggregate particles, will produce spatial frequencies to high resolution. This is called the power score. Parameters for the normalized cross correlation (NCC) and power score to distinguish good particles are shown in Table I. The majority of particles is now rejected. 881 241 particles remain. The particles are finally extracted from the micrographs using the routine “extract from micrographs” in cryoSPARC with a box size of about 500 Å (see Table I), which is much larger than the particle of interest. 650 150 particles are extracted. The particles were sorted into various 2D classes based on their orientation relative to the electron beam. For that, multiple rounds of 2D classification are required. For the first round, we set the number of 2D classes to 100 and the other parameters were kept as default. After the 2D classes were determined, only those classes are retained that appear like 2D classes of the target protein. 13 “junk” classes were excluded. Seven more rounds of 2D classification followed by selection of “good” 2D classes were performed. 107 053 particles were retained. These curated particles are used to generate high-quality, *ab initio* 2D templates. The *ab initio* 2D templates are used to sort again through all curated micrographs (corrected by ice-thickness, etc.) picking particles again aided now by the 2D templates. For this, cryoSPARC provides the routine “template picker” (Table I). 9 539 347 particles were picked and assessed by slightly different parameters based on histograms displayed by cryoSPARC. 586 281 particles populate 27 2D classes based on the 27 different *ab initio* templates. The 2D classes were combined to three 3D volumes using “*ab initio* reconstruction” in cryoSPARC. 3D volumes are obtained with 201 165 particles in class 0, 167 184 in class 1, and 209 932 in class 2. This is done, because the program will otherwise combine all particles, even the sub-quality particles in one class, which may produce a low-quality 3D volume. The 3D classes are subject to “heterogenous refinement.” Here, the images are slightly reoriented to increase the global contrast in the 3D maps. All parameters are kept as default. The quality of the 3D reconstruction can be further increased by “non-uniform refinement” where the particle images were slightly reoriented to increase the contrast locally. Default parameters were used for this process. Class 2 revealed the best overall resolution as estimated by Fourier shell correlation (FSC) [Fig. 3(b)]. The local resolution was determined by the “local resolution estimation” feature in cryoSPARC. The local resolution map of class 2 is displayed in ChimeraX as shown in Figs. 2 and 3(a).

**TABLE I.** Parameters and statistics for cryoEM analysis.

CryoSPARC processing parameters	
General image analysis	Pixel size: 0.829 Å, voltage: 300 kV, spherical aberration: 2.7 mm, and total exposure dose: 45.47 e^−^/Å^2^.
Manual curate exposure	Relative ice thickness (unitless): 1–1.08, and CFT fit resolution: 2–7 Å.
Blob Picker	Particle Ø from 70–220 Å.
Inspect particles	NCC > 0.620, and local power: from −341 to −65 (empirical).
Extract from micrographs	Extraction box size: 600 pix.
2D classification	Initial: 100 classes 7 iterations: 100 classes, batch size per class: 200, initial classification uncertainty factor: 5, and number of online EM-iteration: 40.
Template picker	Particle Ø 195 Å.
Ab initio reconstruction	Number of *ab initio* classes: 3.
Global resolution by FSC	6.0 Å.

### Cryo-EM structure determination

**FIG. 4.**
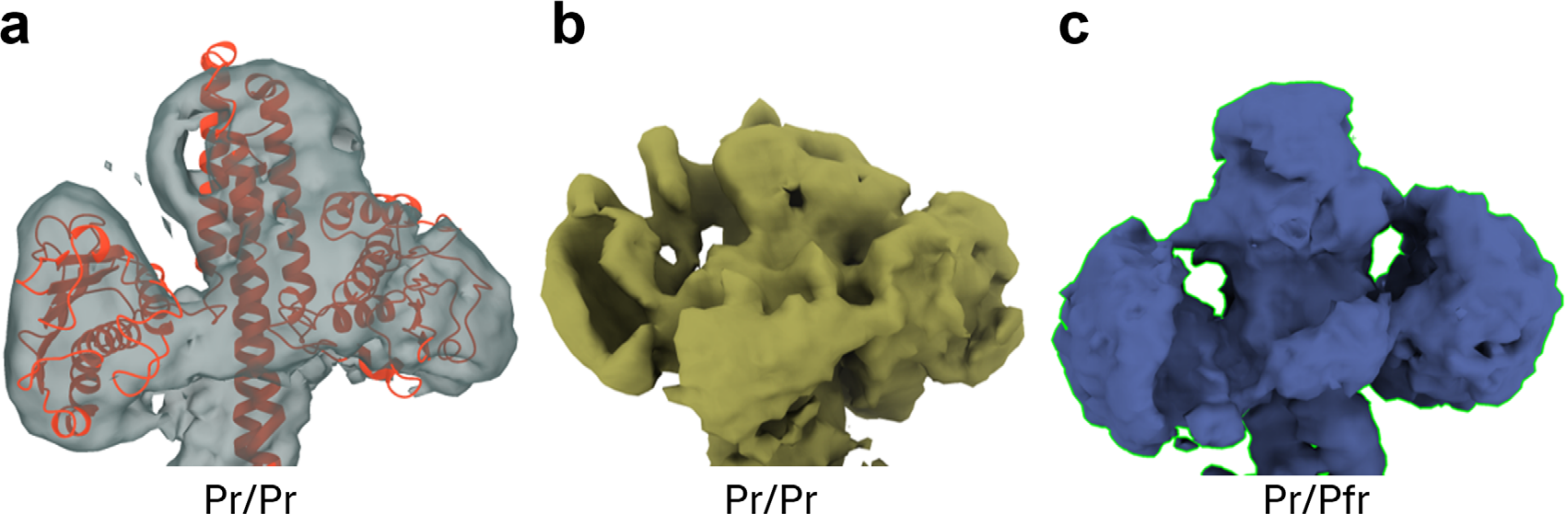
Comparison of the density in the HK region only. (a) Pr/Pr homodimer, this study (with structural model in red), (b) the Pr/Pr density as determined previously (Malla *et al.*, 2024), and (c) the Pr/Pfr heterodimer density (Malla *et al.*, 2024).

Although the cryo-EM map was only around 6 Å, the EM density of the sensory tongue has been good enough to distinguish a β-sheet conformation from two separated strands with one strand transformed to an α-helix [see also Fig. 1(c)]. The density for both subunits agrees with the β-sheet conformation. Accordingly, the density map represents a Pr/Pr homodimer. The map near the HK region is better resolved than the previously published map of a SaBphP2 Pr/Pr homodimer [compare Fig. 4(a) and Fig. 4(b)]. However, Malla *et al.* (2024) reported single-particle cryo-EM structures not only of the SaBphP2 Pr/Pr homodimer, but also structures of a Pr/Pfr heterodimer and a Pfr/Pfr homodimer. Except for the Pr/Pfr heterodimer [Fig. 4(c)], the cryo-EM maps' spatial resolution near the HK region has been too low to model the structures of the HK lobes (Wahlgren *et al.*, 2022; Malla *et al.*, 2024). Here, however, one of the Pr-subunits of the Pr/Pfr heterodimer determined by Malla *et al.* (2025) could be used to interpret one half of the cryo-EM map by placing the Pr subunit into the map by hand. The Pr/Pr dimer was created by duplicating the Pr monomer structure, which was moved by hand in the remaining part of the cryo-EM map. Each subunit can be fit into the density as a rigid unit using ChimeraX (Pettersen *et al.*, 2021). Due to the limited resolution, the structure was not further refined.

### X-ray crystallography

#### Crystals

After the Talon Co^+2^ affinity purification described above for cryo-EM investigations, the SaBphP2 is further purified by size-exclusion chromatography using Superdex S-200 resin. The ultra-pure SaBphP2 is sent to the Hauptman Woodward Institute (HWI, Buffalo, NY) for crystallization trials. The protein tends to crystallize with PEG 20 000 at pH 9 after 2 weeks. One of the conditions identified at HWI can be reproduced in microbatch plates (Hampton Research HR3-081) by mixing 2 *μ*l full-length SaBphP2 (10 mg/ml) with an equal amount of 0.1 M TAPS, 0.1 M Mg(NO_3_)_2_ * 6H_2_O, 24% PEG 20 000 pH 9.0 in drops under oil (silicon oil, Hampton Research HR3-415). Nicely shaped, larger crystals (<50 *μ*m) grew (supplementary material Fig. S1). The crystals were transported within the plate from the United States (Chicago) to the microfocus beamline ID23-2 (Nanao *et al.*, 2022) at the ESRF and survive the journey (bus, flight, taxi) in the plate depressions containing the watery drops. The crystals were scooped out of the drops using MiTeGen loops (Ø 50 *μ*m) and frozen immediately in liquid nitrogen without applying a cryo-protectant.

### X-ray data collection

At ESRF ID23-2, the crystals are exposed to x-rays in an automatic fashion without user interference. Diffraction patterns (supplementary material Fig. S1) are collected by a Dectris EIGER X 9M. The diffraction patterns can also be transferred to the home laboratory for further analysis. Since the EIGER stores the data in HD5-format, we extracted the diffraction patterns in a more commonly accessible format, that of the Dectris PILATUS detector (CBF-format). This was achieved by the program “eiger2cbf” (check www.mrc-lmb.cam.ac.uk/harry/imosflm/ver721/downloads/). The CBF-format files were processed using iMosflm (Powell *et al.*, 2017) and further scaled by programs such as “aimless” (Evans, 2006) included in the CCP4-suite of programs (Winn *et al.*, 2011). In our case, the refined unit cell-constants in an orthorhombic unit cell (later determined as P2_1_2_1_2_1_) are much too small to harbor a full-length SaBphP2 molecule in the asymmetric unit (Table II), and a truncated fragment of the full-length SaBphP2 must have crystallized.

**TABLE II.** X-ray data collection and refinement statistics.

Data collection^a^	
Beamline	ESRF ID23-2
Energy/wavelength	14.2 keV/0.87 Å
Temperature	100 K
Space group	P2_1_2_1_2_1_
Unit cell (Å)	a = 60.8 b = 101.1 c = 103.4
# observations	179 398
# unique	35 675
Resolution range (Å)	52.4–2.1
R_merge_ (%)	8.9 (111.2)
CC_1/2_ (%)	99.4 (61.9)
Completeness (%)	95.6 (97.6)
Signal to noise ratio	7.7 (1.0)
Multiplicity	5.0 (5.0)
Refinement statistics	
Software	Phenix v.1.21
Highest resolution	2.1 Å
# reflections	35 532
R_cryst_/R_free_	22.7/26.0
H_2_O	384

^a^ Highest resolution shell in brackets (2.13–2.1 Å).

### Structure determination

A molecular replacement (MR) is necessary to find the orientation of a SaBphP2 fragment in the new crystal form. The program “phaser” (Oeffner *et al.*, 2013) implemented in “phenix v1.21” (Liebschner *et al.*, 2019) is used. Neither the full-length (Malla *et al.*, 2024) nor the shorter PCM (Sanchez *et al.*, 2019) lacking the HK of SaBphP2 results in a successful MR solution. However, when a fragment consisting only of PAS and GAF domains (the chromophore binding domain, CBD) was tried, a MR solution can be found in space group P2_1_2_1_2_1_. The solution consists of two subunits (A and B) in the asymmetric unit. The MR solution is refined with rigid-body refinement and conventional refinement with “phenix.” Manual intervention is necessary to orient the chromophores of subunits A and B and multiple side-chains into the electron density. There is no electron density for a stretch of amino acids from 115 to 137 that usually forms a helix. Due to space constraints at the dimer interface, this helix is most-likely missing from the construct entirely and is not included in the refinement. The R-free decreases from 34% when the helix was included to 29% after excluding the helix. Refinement statistics is listed in Table II.

## RESULTS AND DISCUSSION

### Cryo-EM structure of the full-length Pr/Pr homodimer

The local resolution map of the 3D reconstruction is shown in Fig. 3(a). The atomic model of the Pr/Pr homodimer is shown by a blue ribbon. The HK lobes occupy positions that are commensurate with the HK orientations observed earlier for a Pr/Pfr heterodimer [Figs. 3(c), 3(d), and 4(c)] (Malla *et al.*, 2024). The local resolution is uniform through the entire SaBphP2 and agrees with the overall resolution. In previous studies (Wahlgren *et al.*, 2022; Burgie *et al.*, 2024), the densities of the HK lobes were all distorted [see, e.g., Fig. 4(b)], except the one of the Pr/Pfr heterodimer [Fig. 4(c)] identified in the ground-breaking study by Malla *et al.* (2024). We speculate that the cryo-EM grid used in the “Spotiton” technique has an influence on the structural heterogeneity, which becomes more uniform compared to conventional grids. Our example shows that cryo-EM density of HK lobes can be obtained for the Pr homodimer. The resulting structure, together with the cryo-EM structures determined by Malla *et al.* (2024), strengthens the plausible signaling mechanism described above: the RR binds to the Pr homodimer, is trans-phosphorylated by the HK (Kumarapperuma *et al.*, 2023), and dissociates from the Pfr homodimer after a substantial HK rotation following photoactivation of the phytochrome. To further investigate the mechanism, binding and dissociation kinetics of the RR to the SaBphP2 must be determined, which should also depend on the various phosphorylation states of the BphP and/or the RR.

### X-ray structure of the SaBphP2 CBD as a fragment of the full-length phytochrome

**FIG. 5.**
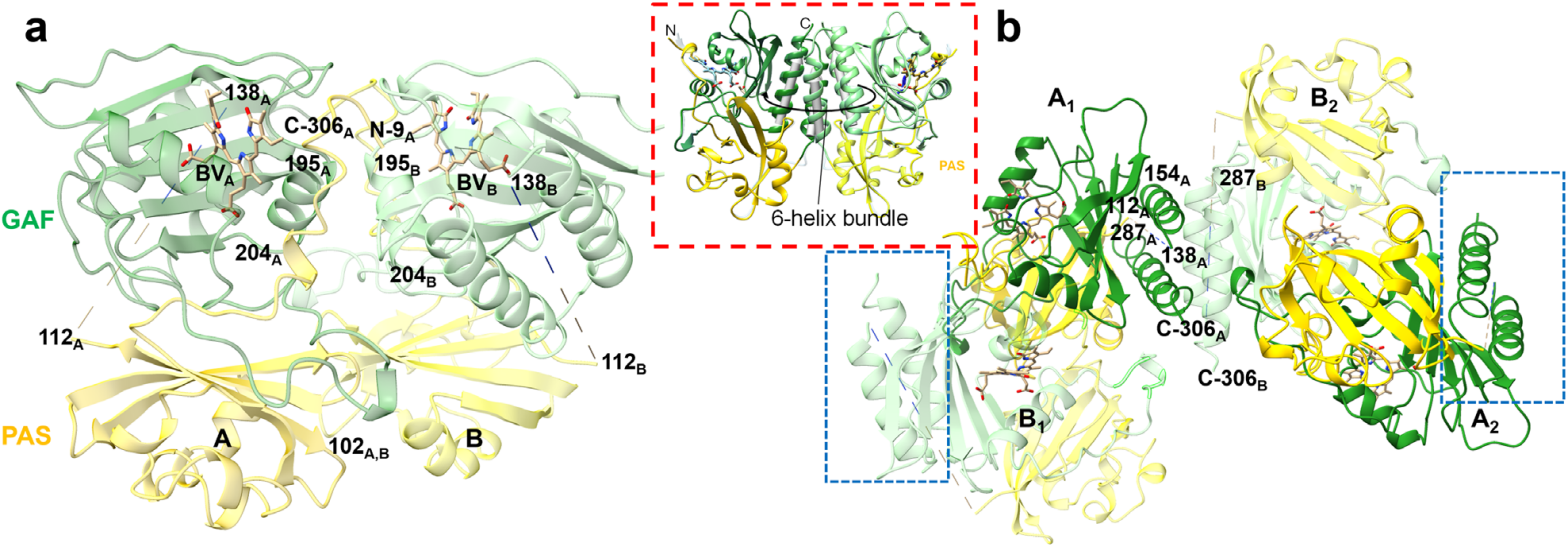
X-ray structure of the truncated fragment of the full-length SaBphP2; overall view: (a) the dimer_NCS_. Interactions between the two NCS related subunits occur between the single helices from amino acid (AA) 195 to 204 (marked). The N-terminus, the C-terminus, and several other structural moieties are marked by residue numbers. The missing helix (from AA 112 to 138) is indicated by a dashed line. (b) Symmetrically equivalent dimer of dimers (DD_sym_). Subunit A is displayed in stronger colors than the B-subunit. Two helices form A and B each contribute to a 4-helix bundle at the subunit interface. The missing stretch of AAs would be located in the space between the helices, which is sterically not possible. The helices of the respective subunits are almost perpendicular to each other. The blue dashed boxes indicate the docking sites for the next four subunits across the crystal symmetry boundaries. Inset (red dashed box): typical dimer interface of a CBD (from Woitowich *et al.*, 2018). A 6-helix bundle can be identified, and the subunits are almost parallel. The helices in the 6-helix bundle are not equivalent to the interface helices in (a) but some can be equated with those in the 4-helix bundle of (b).

The overall structure of the fragment differs from those of the CBD of SaBphP1 and the CBD part of the SaBphP2 PCM. The asymmetric unit harbors a dimer (dimer-NCS) where the subunits are related by non-crystallographic symmetry (NCS) [Fig. 5(a)]. Subunits in the dimer-NCS interact much differently than in the CBDs of the other constructs. A dimer of dimers (dimer-SYM) is formed through crystallographic symmetry [Fig. 5(b)]. The dimer-SYM interface is formed by four helices equivalent to those of the SaBphP1 CBD. However, the angle between the interface helices is about 90° rather than around 35° as observed for the other constructs mentioned. The interfaces of both the dimer-NCS and the dimer-SYM are very different from those normally observed in CBD and PCMs of BphPs (Woitowich *et al.*, 2018). It appears as NCS and crystallographic symmetry that alternate with each other, a supermacromolecule (with a very large number of subunits) is formed that stretches through the entire crystal [Fig. 5(b), dashed boxes]. It is very well conceivable that such a truncated construct, if even stable, when used in crystallization attempts, would never crystallize and would form amorphous adducts.

**FIG. 6.**
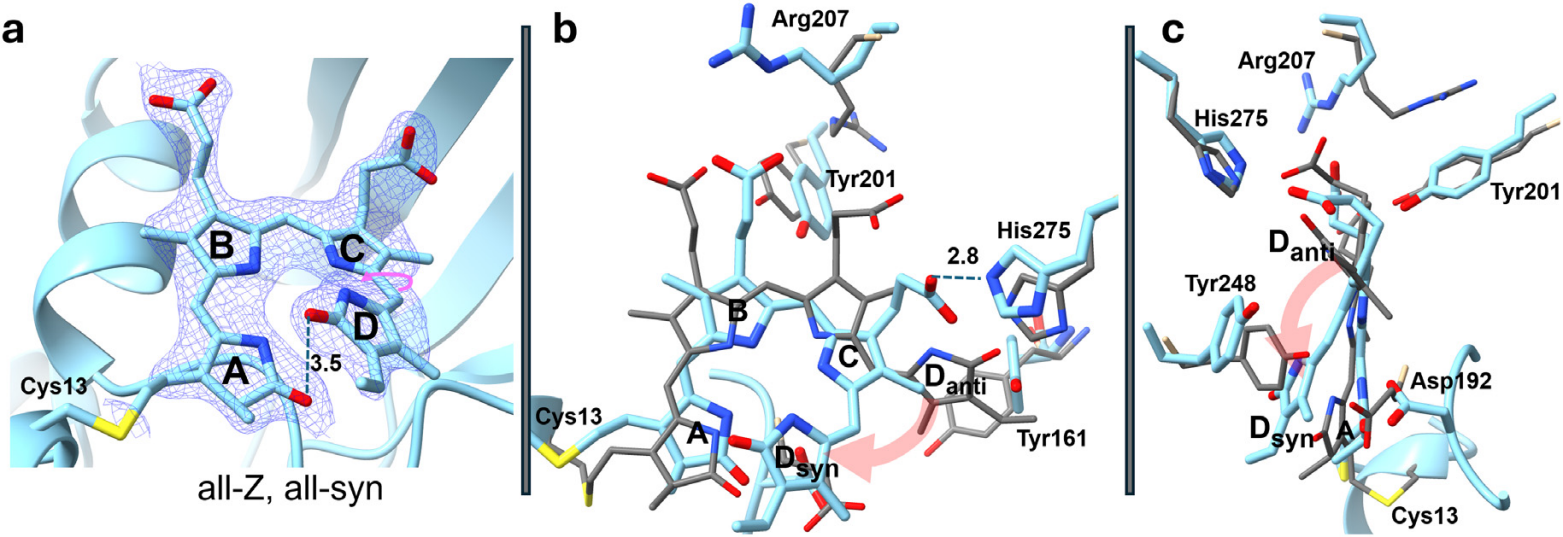
The chromophore pocket. (a) Electron density of the chromophore. Blue contours: 2mFo-DFc electron density map of the BV chromophore (1.4 σ contour level). The chromophore pocket and the BV chromophore (with pyrrole rings A–D) in the all-syn configuration are shown. The ring-D rotation is denoted by a pink arrow. The ring-A and ring-D oxygens are 3.5 Å apart. (b) and (c) Overlay of amino acids of the fragment determined in this study (blue) on selected amino acids (grey); a SaBphP2-PCM reference structure determined earlier (pdb-entry 6PTX, Sanchez *et al.*, 2019). The pink arrow shows the displacement of ring-D from the anti (D_anti_) to the syn (D_syn_) configuration. Pyrrole rings A to D are marked. (b) Front view. Large displacements are observed and marked for several amino acid residues. His275 forms a H-bond (2.8 Å) with the ring-C propionyl. (c) Side view of the same pair of structures as displayed in (b), instead of Tyr161 as in (b), Tyr248 is shown.

The most notable difference to all other phytochromes, however, is the structure of the BV chromophore. Rather being in a stretched-out (all-Z) syn–syn–anti configuration, the BV assumes a curved all-Z, all-syn configuration [Fig. 6(a)], which has never been observed so far. BV originates from heme, which can be regarded as a closed ring, however also in the all-Z, all-syn configuration. However, BV cannot close to heme again, as this requires a reduction reaction. Consequently, ring-D is stacked on top of ring A, and the BV forms a spiral staircase [with the pyrrole rings as four steps, Fig. 6(a)] in both subunits. The pitch of the staircase is 3.5 Å [see the distance of the ring-D oxygen to that of ring-A in Fig. 6(a)].

The configurational change of the BV results in numerous large structural changes of nearby amino acid residues. Some of the largest structural displacements are shown in Figs. 6(b) and 6(c). In order to assume the all-syn configuration, the BV must rotate and flip ring-D about the 14–15 single bond to the ring-D_syn_ position [Figs. 6(b) and 6(c)]. As a result, Tyr248 moves away [Fig. 6(c)] and Tyr161 can now occupy the space of ring-D_anti_ [Fig. 6(b)]. His275 now forms a hydrogen bond with the ring-C propionyl, which is reminiscent of a Pfr structure [Figs. 6(b) and 6(c)]. Tyr201 moves in between the ring-C and ring-D propionyls [Fig. 6(b)]. As a result, Arg207 moves away from its constrained position and stretches out [Figs. 6(b) and 6(c)].

### Cryo-EM with “Spotiton” and x-ray crystallography with full-length SaBphP2

Our early results with Spotition encourage time-resolved experiments. For this, illumination conditions must be found that drive the phytochrome from the Pr into the Pfr state on the grid. After initial attempts with a high-power diode that did not result in a substantial fraction of the molecules to transition into the photocycle, we will now install a higher power laser in the “Spotiton” device. We expect that, based on the absorption cross section of the phytochromes, the ensemble is effectively driven into the photocycle. By optimizing the size of the drops containing SaBphP2 sprayed on the Spotiton nanowire grid, we expect an improvement of spatial resolution compared to that achieved here. Nevertheless, even with 6 Å spatial resolution, and >20 ms temporal resolution, heterodimer formation and subsequent HK domain rotation will likely be observable in real time.

A near-atomic resolution x-ray structure of a full-length phytochrome with HK activity might come in reach but has not achieved here. Currently, with x-rays, we are limited to the investigations on the shorter PCM at various time scales (Carrillo *et al.*, 2021; Malla *et al.*, 2025), which might be plagued by kinetic artifacts due to crystal lattice constraints (Malla *et al.*, 2024). For the full length BphP, other crystallization conditions at perhaps lower pH and temperatures must be found that do not result in protein fragmentation and in corresponding structural artifacts. Nevertheless, the fragmentation results in an interesting chromophore configuration and large amino acid displacements that were never seen before in phytochromes and that could even be transiently occupied at some stage in the photocycle.

## Data Availability

The X-ray structure of the SaBphP2 fragment and the corresponding structure factor amplitudes have been deposited into the protein data bank with accession code 9NAA. The cryo-EM map has been deposited into the EMDB with entry EMD-70119.
